# Work, Play, and Success

**Published:** 1890-09-13

**Authors:** A. Pearce Gould

**Affiliations:** Senior Assistant-Surgeon, Middlesex Hospital


### Work, Play, and Success.

By A. Pearce Gould, M.S., F.R.C.S., Senior Assistant-Surgeon, Middlesex Hospital.

In venturing to respond to the editor's request that I would write a short article specially addressed to medical students to appear in the "Students' Number" of The Hospital, I purposely refrain from any attempt to exalt the medical profession either on account of its inherent nobility, for the great work it accomplishes, for the grand names connected with it, or for the prospect it offers of a competent livelihood. Nor shall I discuss the details of the present curriculum with the intention of indicating its merits or its faults. But I shall content myself with offering a few very simple, even common-place, fragments of advice to those who have already determined to follow the profession of medicine, and to whom an essay on the choice of a profession would be of no immediate practical interest. If what I say appears very trite to my readers, I must beg them to bear in mind that to give good advice is only less difficult than to act upon it, and that just as our lives are mainly influenced by circumstances which at the time appeared trivial and not by great crises, so a student's successful course may often be due to attention to what at the time seem minor matters.

Undoubtedly, the most important point for a student to keep always in view is that his work at his school and hospital is a preparation for his life's work, and that upon it depends, to a very large extent, his future prosperity. To realise this will, of course, make a man earnest in his work, persistent, and painstaking; but it ought to do more than that. It should stimulate him to make his foundation broad and solid. His knowledge must be accurate, not ill-defined, and general, if it is to be applied in after years. He must grasp the principles and fundamental laws of the branches of natural science or of medical and surgical practice that he studies, rather than learn off a great number of isolated facts and rules. And further, he must maintain a solidarity in all his work. He will attend classes in which separate subjects are taught, and his different teachers will sharply limit their range of instruction. The student must, however, remember that this is largely artificial and merely the result of the necessity for the division of labour, and he must correct it in his own mind, and be careful to weld all branches of his studies into one great whole. His work will then be not only more effective and more real, but it will have a charm not otherwise attainable. And to work in this way will dissipate two very common fallacies, for it will show the absurdity of regarding any study as of merely temporary importance, and it will help to assist the student to estimate rightly the importance of examinations. The mapping out of the medical curriculum in such a way that particular subjects are studied at certain definite periods only, and an examination in one subject or set of subjects having to be passed before the student can proceed with the next subject in his long list, of necessity, begets the notion that these different studies may be regarded as the rungs of a ladder rather than as the links in a chain. The fact is that no one of the many subjects in the medical curriculum is separate from the others, a thing to be considered complete in itself, an independent entity. But all are as mutually dependent as the separate links in a chain. Perhaps it is even more essential thoroughly to realise that the one supreme end of all knowledge is not the passing of an examination. The multiplication of examinations and the splitting of them up into sections which can be separately passed, makes the student very prone to exaggerate their importance, and to work more with the object of passing them than to prepare himself for his life's work. I would not undervalue examinations, nor have my readers in the least indifferent to their importance. They must be passed, and the sooner the better; but let them be merely incidents in the student's course, not the goal itself. For instance, a man who learns his anatomy simply that he may pass his second professional examination can easily succeed in his aim, but his knowledge will soon pass from him, and he will be little, if at all, the better for it when he wants to practice his profession. Only one other remark on this point lest I labour it too much. Students should be very careful to master so far as they can the various instruments by which clinical and pathological investigations are made, for this enables them afterwards continually to add to their knowledge. For instance, a student should be most careful to learn how to use the microscope and how to prepare objects for it, how to use the ophthalmoscope, laryngoscope, and stethoscope, and should as far as possible acquire familiarity with all the methods of diagnosis. Such knowledge will stand him in far better stand in after life than much book knowledge or a superficial acquaintance with rare forms of disease. I would repeat what I have already said that a student's career is successful only so far as it is a sound preparation for the practice of his profession, and that success can in no case be rightly estimated by prizes won or by examinations successfully passed.

And now a word or two as to recreations, a subject constantly discussed, and on which extremely different views are taken. I will not assume that any of my readers would dispute the need for recreation, and I will limit myself to stating what I hold to be the one essential condition of recreation, and to offering one piece of advice concerning it. The essential condition to be observed in any recreation is that it shall be a help and not a hindrance to a student's work. We are too apt to think that a thing good in itself cannot be a cause of evil; whereas its influence upon us wholly depends upon the use we make of it. Let me illustrate my meaning by a simple case. Football is now the favourite sport with medical students, and I am convinced that it is at one and the same time a source of great good and of great evil to many in our medical schools. To those who play a good game on a Saturday afternoon, and then work harder all the succeeding week because of the invigorating effect of the active physical exercise, it is an unmixed good. But I think I have known men who have become so absorbed in their game on Saturday that they have been unable to banish it from their minds on Monday and Tuesday, and then on Thursday and Friday their thoughts have been full of the next match. These men suffer from their sport. Yet, at any rate, they have the advantage of the active physical exertion. But others there are who never play a game and content themselves with watching their fellows, and spend much of the following week in talking over the incidents of the game, and apparently find great enjoyment in the newspaper reports of matches. To such the game is an unmixed evil. A man should work while he works, with all his faculties fully intent on the matter in hand. And then when he plays he should play, and not merely think and talk about it, or watch others at play.

But even in his recreation a student should prepare for his future life. So far as possible he should cultivate some pursuit which he will be able to follow up in after years, and to which he can turn aside for refreshment and delight when wearied by the toils of a busy practice. In the choice of such a recreation much depends upon the man's taste, much also upon his powers. Some will turn to one of the many fields of biology which lie outside the general medical curriculum; others may choose some other branch of natural science; others again will turn to art, or music, or archeology, or history, or poetry. But whatever it may be, a really good recreation that can be pursued and enjoyed throughout life is a most valuable addition to the resources of a man's life.

And now I must bring these remarks to a close, but not without emphasising the fact that in no department of life is it more essential than in the profession of medicine to remember that "character makes the man." This partly arises from the intimate relations into which it brings a man with his fellows of all ranks and in all circumstances. Partly it springs from the fact that in no other department of life are the opportunities for practising dishonesty in its various, more or less specious, forms more numerous or more difficult to guard against. And to win the highest and most worthy success a student must not only work well and wisely, and use recreations as a help to his work, but he must learn to maintain "a conscience void of offence toward God and man."

